# Neoadjuvant Imatinib Therapy for Gastrointestinal Stromal Tumors Associated With Non-islet Cell Tumor Hypoglycemia (NICTH): A Case Report

**DOI:** 10.7759/cureus.65903

**Published:** 2024-07-31

**Authors:** Sawsen Nouira, Ines Bayar, Ekram Hajji, Marmouch Hela, Ines Khochteli

**Affiliations:** 1 Endocrinology and Diabetes, Farhat Hached Hospital, Sousse, TUN; 2 Endocrinology, Faculty of Medicine, Fattouma Bourguiba University Hospital of Monastir, Monastir, TUN

**Keywords:** thyrosin kinase inhibitor, surgery, non-islet cell tumor hypoglycemia, imatinib therapy, gist

## Abstract

Non-islet cell tumor hypoglycemia (NICTH) is a rare paraneoplastic syndrome characterized by insulin-like growth factor-2 (IGF-2) release, often associated with diverse tumor types. Gastrointestinal stromal tumors (GISTs), sarcomatous lesions of the gastrointestinal tract, are rarely associated with NICTH.

We present a unique case of a 58-year-old patient diagnosed with a GIST exhibiting recurrent hypoglycemia suggestive of NICTH. Despite normal IGF-2 levels, the IGF-2/IGF-1 ratio supported the NICTH diagnosis, which was confirmed histologically. Imaging revealed a large intraperitoneal mass. Hypoglycemia was managed with high-dose dextrose and hydrocortisone. Treatment with the tyrosine kinase inhibitor, imatinib, was initiated. Surprisingly, imatinib not only reduced the tumor size but also improved hypoglycemia. The study highlights the complexities in managing NICTH and its underlying causes. Current diagnostic limitations, treatment modalities, and unexpected therapeutic responses challenge standard approaches. This emphasizes the need for personalized oncological strategies.

## Introduction

Non-islet cell tumor hypoglycemia (NICTH) is a rare paraneoplastic syndrome characterized by the release of insulin-like growth factor-2 (IGF-2) and can be instigated by diverse tumor types [1,2]. NICTH was observed in a total of 130 cases across 98 case reports or small series (each involving fewer than 10 patients) within the English medical literature from January 1, 1988, to August 15, 2013 [3]. Its occurrence is estimated at approximately one per million person-years [4]. Tumors associated with NICTH produce peptides, cytokines, and growth factors, including IGF-2, contributing to the development of hypoglycemia.

In cases of NICTH associated with certain tumors, particularly GISTs, there can be an overproduction of a variant of IGF-2 known as "big" IGF-2 which is characterized by its inability to bind to IGFBP-3 and the acid-labile subunit to form the ternary complex [2]. As a result, "big" IGF-2 circulates in its free form, leading to several biochemical and physiological effects that contribute to hypoglycemia, such as impaired glucose release from the liver, increased glucose utilization, and suppression of counter-regulatory hormones [2].

NICTH, due to its infrequency, atypical clinical symptoms, and inconclusive laboratory results, may be prone to misdiagnosis. It can lead to prolonged and severe hypoglycemia until surgical tumor removal, potentially influencing tumor management strategies [5]. Tumors linked to NICTH encompass a spectrum from benign to low-grade malignant and high-grade malignant tumors. This range includes hepatocellular carcinoma, lymphoma, and various mesenchymal tumors [1,4].

Gastrointestinal stromal tumors (GISTs) are sarcomatous tumors of the gastrointestinal tract, primarily located in the stomach, small intestine, and large intestine [6]. We present an unusual case in which a patient diagnosed with a malignant GIST and hepatic metastases exhibited an initial manifestation of hypoglycemia, suggestive of NICTH. Our case underscores the challenges encountered in managing both the hypoglycemia and the tumor.

## Case presentation

A 58-year-old male was transferred from the psychiatric department due to recurrent hypoglycemia. He had no significant family history. He had a history of COVID-19 infection one year before, which did not require hospitalization. There were no chronic medications being taken prior to the onset of symptoms. Two months prior to hospitalization, the patient presented symptoms of anxiety and depression. Two months later, he experienced early morning confusion. Neurological concerns were ruled out. Subsequently hospitalized in psychiatry due to depressive symptoms, the patient displayed recurring hypoglycemic episodes mainly outside mealtimes. Glucose level monitoring indicated levels between 30 and 40 mg/dL. The patient was transferred to the endocrinology department. Functional symptoms revealed loss of appetite, fatigue, weight loss, and constipation.

His body mass index (BMI) measured 28 kg/m². He had normal blood pressure, 110/60 mmHg. Abdominal examination indicated dullness upon percussion. The laboratory analysis performed to investigate hypoglycemia revealed reduced levels of insulin and C-peptide (Table 1).

**Table 1:** The laboratory findings of the patient. IGF-1: insulin-like growth factor 1; IGF-2: insulin-like growth factor 2

Laboratory findings levels	Patient	Normal range
Insulin	1.1 IU/mL	1.9-23 IU/mL
C-peptide	0.2 ng/mL	1.1-4.4 ng/mL
IGF-1	18 ng/mL	54-194 ng/mL
IGF-2	509 ng/mL	288-736 ng/mL
IGF-2/IGF-1 ratio	28	-

**Figure 1:**
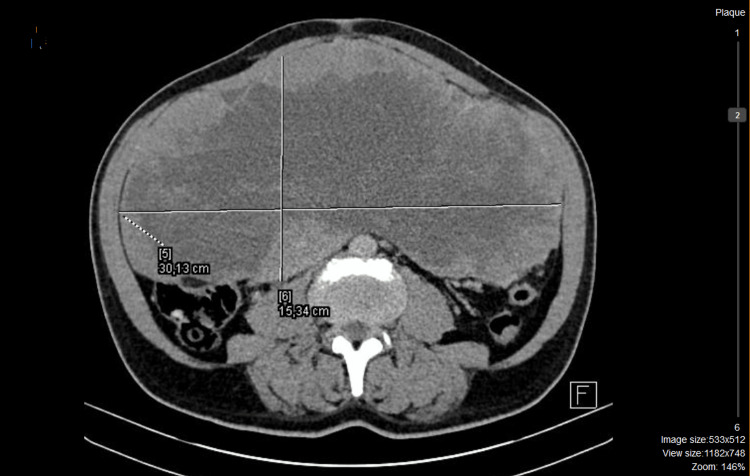
Abdominal computed tomography (CT) imaging revealing a 30x16x26 cm abdominal mass before treatment with imatinib (axial section).

The screening for sulfonylurea yielded negative results. There was a notable suppression in insulin-like growth factor 1 (IGF-1). A computed tomography (CT) of the chest and abdomen detected a substantial intraperitoneal mass measuring 30x16x26 cm arising from a small bowel loop. The mass displayed irregular enhancement with areas of central necrosis, exhibiting anatomical connections with major blood vessels (Figure 1).

**Figure 2:**
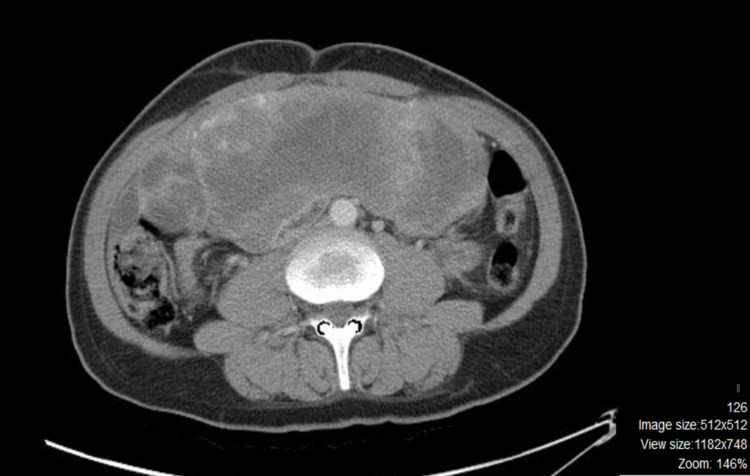
Abdominal computed tomography (CT) imaging after six months of treatment with Imatinib revealing a decrease in the size of the tumor (25x14x22 cm) (axial section).

Persistent hypoglycemia required continual management using high concentrations of dextrose solution and repeated administrations of hydrocortisone. A core biopsy was conducted, confirming that the pathology was consistent with a gastrointestinal stromal tumor (GIST). Surgery was not feasible. The patient was started on the tyrosine kinase inhibitor (imatinib). After six months of treatment, a follow-up assessment showed a decrease in the size of the tumor mass by 15% (25×14×22 cm) (Figure 2).

Over time, there was an improvement in the patient's blood glucose levels with the use of imatinib. As a result, the requirement for hydrocortisone and glucose solution decreased, eventually leading to their discontinuation due to the absence of hypoglycemia. The treatment was extended for six months. After 20 months of regular treatment with imatinib, a reassessment of the tumor size revealed a remarkable reduction. Following a multidisciplinary decision, the patient underwent surgical removal of the tumor, experiencing a straightforward postoperative recovery, and achieving normalization of glycemia without the need for further treatment.

## Discussion

This study indicates that reduced insulin levels coupled with hypoglycemia strongly suggest a diagnosis of NICTH as a paraneoplastic syndrome associated with GIST. The yearly GISTs have been documented to range from 6.8 cases per million in the United States to 14.5 cases per million in Sweden [7]. Typically, these tumors are prevalent in the stomach (40-70%), followed by the small intestine (20-40%), with less than 10% occurring in the esophagus, colon, and rectum [8,9].

Into the 1970s and early 1980s, the precise mechanism through which NICTH triggered hypoglycemia remained ambiguous. It was during this time frame that researchers identified circulating insulin-like peptides, offering crucial insights into these mechanisms [10,11]. In situations where NICTH is linked to GIST, the tumor's production of a modified insulin-like growth factor (IGF)-2 is the underlying cause of the hypoglycemic episodes.

This variant of IGF-2, known as "big" IGF-2, is labeled as such due to its molecular weight ranging between 11 kDa and 18 kDa, contrasting with the standard IGF-2 molecular weight of 7.5 kDa [12]. Under normal conditions, IGF-2 typically forms a ternary complex with insulin-like growth factor-binding protein (IGFBP)-3 and a labile acid subunit. However, "big" IGF-2 lacks the capacity to create this complex. Consequently, there's a reduction in glucose release from the liver, coupled with an increase in glucose consumption by skeletal muscle, leading to hypoglycemia [13]. Additionally, "big" IGF-2 suppresses the release of growth hormone, glucagon, and the production of IGFBP-3 [14]. This collective effect suggests that "big" IGF-2 contributes to the sustained hypoglycemia observed in (NICTH) [15].

In addition to hypoglycemia, these tumors manifest through various symptoms, including abdominal pain with a palpable mass in the abdomen, gastrointestinal bleeding, anemia, nausea, vomiting, constipation, diarrhea, and weight loss [16]. Detecting asymptomatic GISTs typically presents a diagnostic challenge and is often incidentally found through imaging techniques like CT scans or endoscopy.

Understanding hypoglycemia requires evaluating serum levels of glucose, insulin, proinsulin, and C-peptide. The Endocrine Society Clinical Practice Guideline provides specific fasting plasma level thresholds for laboratory testing in non-diabetic hypoglycemia as follows: insulin level <3.0 mmol/L, proinsulin level >5.0 pmol/L, and C-peptide level >0.2 nmol/L [16]. Essential additional tests involve examining IGF-1, IGF-2, and growth hormone (GH) levels. Notably, no commercially available assay currently measures "big" IGF-2, a crucial diagnostic marker for NICTH. Subsequently, the diagnostic path often includes cross-sectional imaging of the chest, abdomen, and pelvis [3].

GIST diagnosis involves histological analysis, often confirmed via immunohistochemical staining for CD117 (c-KIT), which is positive in around 95% of GIST cases [2,6]. The treatment options for NICTH, particularly non-metastatic versus metastatic cases, differ based on the extent and feasibility of surgical intervention [3].

For non-metastatic tumors, surgical resection, debulking, or tumor embolization are effective treatments. Complete surgical removal is often the most definitive approach, especially for tumors causing hypoglycemia, which can be resolved upon complete excision. However, complete resection might not always be possible due to reasons such as large tumor burden, extensive metastatic spread, patient preference, or tumor location in relation to critical structures.

GISTs typically show resistance to traditional chemotherapy. However, the survival rates for metastatic GIST have seen significant enhancement since 2002, following the approval of tyrosine kinase inhibitors like imatinib mesylate by the US Food and Drug Administration (FDA) [17]. The fast-track approval stemmed from a multicenter, randomized study involving 147 individuals with advanced GIST, assessing imatinib's safety and effectiveness at two different daily doses (either 400 mg or 600 mg). The study revealed a clinical benefit rate of 81% for imatinib [17].

Imatinib is effective in cases where surgical resection isn't feasible, particularly for metastatic and unresectable tumors. Imatinib is considered the first-line agent for treating GIST and has shown efficacy in managing such tumors [17].

A pivotal breakthrough in treating GISTs emerged with the understanding of their molecular mechanisms, primarily involving gain-of-function mutations in either the v-kit Hardy-Zuckerman 4 feline sarcoma viral oncogene homolog (KIT) [18] or platelet-derived growth factor receptor alpha (PDGRFA) genes [19,20]. Approximately 60-80% of GIST cases exhibit a mutation in KIT, while around 5-15% have a PDGRFA mutation [1,17]. These mutations lead to the independent activation of kinases, considered the primary catalysts in the development and sustenance of GISTs. GISTs that lack mutations in KIT or PDGRFA are termed "wild-type GISTs" and are less commonly encountered [17].

Prior to surgical removal, hypoglycemia is managed by increasing food intake and sometimes administering intravenous glucose or dextrose. The most effective approach for treating NICTH involves using glucocorticoids, which suppress the production of IGF-2, thereby addressing the hypoglycemia caused by "big" IGF-2 [1,7]. Initiation of glucocorticoid therapy should commence with the smallest feasible dose.

Although existing literature indicates that tyrosine kinase inhibitors (TKIs) typically contribute to reduced blood glucose levels, our patient's response to imatinib treatment has notably improved the severity and frequency of their hypoglycemic episodes. This unexpected outcome contradicts the anticipated effect of TKIs on glycemic levels, suggesting a unique or atypical response to imatinib in this specific case. Further investigation and analysis are warranted to understand the underlying mechanisms responsible for this atypical response and to potentially explore its implications for managing hypoglycemia in similar cases. While our study provides insights into the management of GIST-associated NICTH with imatinib, we acknowledge the limitations inherent in single-case studies. Future research efforts should focus on larger cohorts to validate our findings and elucidate the underlying mechanisms driving treatment response variability.

## Conclusions

Treatment strategies for NICTH-associated GISTs vary based on tumor extent. Surgical intervention remains the primary choice for non-metastatic tumors, while tyrosine kinase inhibitors (TKIs) like imatinib have revolutionized the management of metastatic or unresectable GISTs. While this case aligns with existing knowledge on NICTH and GISTs, the unexpected response to imatinib prompts a need for deeper exploration into individualized treatment responses and their implications for managing hypoglycemia in similar cases, underscoring the importance of personalized approaches in oncology.
